# Special Issue on the Effects of Prenatal Smoking/Nicotine Exposure on the Child’s Health

**DOI:** 10.3390/ijerph18105465

**Published:** 2021-05-20

**Authors:** Mikael O. Ekblad, Julie Blanc, Ivan Berlin

**Affiliations:** 1 Department of General Practice, Institute of Medicine, Turku University and Turku University Hospital, 20014 Turku, Finland; 2 Department of Obstetrics and Gynecology, North Hospital, APHM, Chemin des Bourrely, 13015 Marseille, France; julie.blanc@ap-hm.fr; 3 EA3279, CEReSS, Health Service Research and Quality of Life Center, Aix-Marseille University, 13284 Marseille, France; 4 Department of Pharmacology, Assistance Publique-Hôpitaux de Paris, Sorbonne University, 75013 Paris, France; ivan.berlin@aphp.fr; 5 University Centre of General Medicine and Public Health, 1011 Lausanne, Switzerland

Smoking increases the risk of negative pregnancy and perinatal outcomes and may have negative effects on a child’s short and long-term health. The occurrence and severity of negative postnatal health outcomes may be associated with the prenatal duration of exposure but may also vary according to the timing of exposure, i.e., whether it occurred during the first, second or third trimester only or during the whole pregnancy. In recent years, knowledge on epigenetic programming by smoking exposure during pregnancy has emerged [1]. Epigenetic mechanisms may explain the pathways for the potential first generational and transgenerational effects, i.e., how grandmaternal smoking during pregnancy may influence a grandchild’s health.

Maternal smoking during pregnancy is a special type of second-hand smoke exposure; it exposes the fetus to thousands of toxic compounds from fecundation until the end of pregnancy. The prevalence of smoking during pregnancy largely varies by country and by geographical regions, the highest being in Ireland (38.4%) and the lowest in Tanzania (0.2%) [2]. The prevalence is even higher in certain groups, such as among teenagers, of which over 50% smoke during pregnancy [3].

This Special Issue aimed to bring together the latest research on the influence of prenatal smoking/nicotine exposure on the child’s health. The main findings of the articles of this Special Issue are presented in Table 1.

Low birth weight is known to predispose the child to various future chronic negative health conditions. Smoking is one of the main preventable risk factors for low birth weight (LBW), i.e., birth weight ≤ 2500 g. Ghimire et al. [4] investigated the effect of timing of maternal smoking cessation during pregnancy on the risk of LBW. The authors used routinely collected perinatal data on singleton live births for the period 2011–2019 in Southern New South Wales, Australia. The study population included women who reported smoking during pregnancy (*n* = 2099), of which 17% quit smoking during the second half of pregnancy. Compared to women who smoked throughout pregnancy, those who stopped smoking during the second half of pregnancy had 44% lower odds (multivariate adjusted OR: 0.56, 95% CI: 0.34 to 0.94) of having a baby with LBW. Even mothers who smoked 1 to 10 cigarettes a day during the second half of pregnancy had significantly higher odds (OR: 1.75, 95% CI 1.04 to 2.96) of LBW than mothers who stopped smoking by the second half of pregnancy. These data suggest that stopping smoking even at mid-pregnancy may reduce the risk of LBW and consequently the risk of negative perinatal and postnatal health outcomes.

The paper by Rumrich et al. [5] sheds new light on the transgenerational effects of grandmaternal smoking during pregnancy on the newborn’s body size and proportions by using data from the Finnish birth register 1991–2016 (*N* ≈ 24,000). Smoking by the grandmother during her pregnancy but no smoking of her daughter during pregnancy did not associate with the child’s body size and proportions. However, when both the grandmother and the mother smoked during their pregnancy, the newborns had lower LBW and poorer body size and proportion characteristics than newborns of nonsmoking grandmothers and nonsmoking mothers. Moreover, smoking during pregnancy by both grandmother and mother was associated with significantly poorer birth characteristics compared to maternal smoking only. Thus, the grandmother’s smoking during her pregnancy adds an excess risk to that of maternal smoking for birth characteristics. This paper suggests a new risk factor for potential postnatal negative health outcomes in the child. The increased risk associated with grandmaternal smoking during pregnancy, along with maternal smoking during pregnancy, may act through smoking-induced low birth weight [9].

Smoking exposure has been linked robustly with externalizing problems which are characterized by rule breaking, hyperactive, and aggressive behavior, whereas internalizing behavior is characterized by withdrawn, anxious, and depressed behavior. Recent genetically informed studies have proved that the association is not as straightforward as thought, and it is important to use rigorous settings that control for familial and genetical confounds. Additionally, previous studies have not been able to account for the comorbidity of externalizing and internalizing symptoms. Ekblad et al. [6] studied the association between maternal smoking and severity and directionality of externalizing and internalizing symptoms in a sample of sibling pairs. They used data from The Missouri Mothers and Their Children Study, which included 173 families with sibling pairs who were discordant for exposure to smoking. Maternal smoking during pregnancy was not associated with the combined severity of both internalizing and externalizing symptoms, but it was associated with direction of symptoms toward externalizing symptoms. Their findings support the potential causality between maternal smoking during pregnancy and the child’s externalizing behavior.

This Special Issue also includes two reviews. A narrative review by Nakamura et al. [8] describes the epigenetic regulation mechanisms and review the epigenetic marks in different tissues in association with maternal smoking during pregnancy as well as the multiple technical and methodological challenges regarding the data analysis. The review reports on previous studies demonstrating association between maternal smoking during pregnancy and increased risk of the child being overweight or obese, having attention-deficit hyperactivity disorder (ADHD), substance use disorder, reduced lung function, or impairment of the immune system. The authors provide evidence that maternal smoking associated with DNA methylation changes persist during childhood. Nakamura et al. [7] speculate that epigenetic mechanisms could mediate the observed associations between environmental exposures, such as maternal smoking during pregnancy, and later offspring health outcomes but further findings should strengthen this relationship. Furthermore, these effects may transfer beyond the next generation or even beyond. The authors emphasize, however, that alterations in epigenetic signaling and the role of these epigenetic mechanisms is not yet fully understood.

Nicotine replacement therapies (NRT) are approved medications in some countries to help pregnant smokers who are not able to quit smoking otherwise, cease smoking but the knowledge of the long-term safety and efficacy of NRT use during pregnancy are sparse. Blanc et al. [8] reports on the available knowledge about the postnatal effects of NRT, used during pregnancy, on the child’s health outcomes. Although this issue seems to be of importance, only very few (*N* = 5) papers addressed it and mainly secondarily. The available findings are of low certainty; therefore, no firm conclusions can be drawn. Blanc et al. [9] concluded that there is urgent need for well-designed controlled clinical trials which include a control group of women with no exposure to nicotine, to assess the postnatal safety profile of NRT administered during pregnancy.

This Special Issue did not receive submissions of any studies investigating the effects alternative nicotine delivery systems (ANDS), such as electronic cigarettes, used during pregnancy, on the child’s health. Many pregnant smokers and health care professionals believe that using ANDS is safer than cigarettes. However, the US Preventive Services Task Force identified no studies that addressed the benefits or harms of ANDS to help pregnant women quit smoking [10] and to the best of our knowledge there is no report on the postnatal effects of ANDS used during pregnancy on the child’s health.

In conclusion, the present Special Issue gathered five peer-reviewed and open access articles that provide new insights and valuable information on the effect of prenatal smoking/nicotine exposure on the children’s health. Maternal smoking during pregnancy is undeniably harmful to the mother–child dyad, and the negative effects of prenatal smoking exposure seem to be long-lasting and may even transfer beyond generations. Further, well-powered studies are needed to quantify the public health contribution of prenatal smoking exposure, as well as nicotine exposure, to postnatal negative health outcomes. These studies should distinguish the postnatal effects of the direct prenatal, maternal smoking exposure from the indirect prenatal smoking exposure, i.e., the postnatal effects of secondhand smoke exposure of the non-smoking pregnant women on the child’s health. It is important to use all cessation efforts to prevent smoking, or even eradicate smoking, during pregnancy.

## Figures and Tables

**Table 1:** Main findings of the articles published in this Special Issue.

Authors	Main Focus	Main Findings
Original articles		
Ghimire et al. [4]	Timing of smoking exposure on low birth weight	Women who quit smoking during the second half of pregnancy had 44% lower odds of having a baby with a low birth weight compared to those who smoked throughout pregnancy.
Rumrich et al. [5]	Transgenerational effect of smoking on the newborns birth characteristics	Smoking during pregnancy by both grandmother and mother results in higher odds of the newborns having lower body size and proportionality. Grandmaternal smoking during pregnancy adds an excess risk to maternal smoking during pregnancy for negative birth outcomes.
Ekblad et al. [6]	Maternal smoking and externalizing symptoms in a sibling design	Maternal smoking is associated with differentiation of symptoms toward externalizing symptoms rather than internalizing symptoms in a sibling design study that further controlled for genetic and familial factors. The findings support a potentially causal relationship between smoking during pregnancy and externalizing behavior.
Reviews		
Nakamura et al. [7]	Maternal smoking and epigenetic alterations	The review describes the epigenetic regulation mechanisms and reviews the epigenetic marks in different tissues in association with maternal smoking during pregnancy as well as the multiple technical and methodological challenges regarding epigenetic data analyses.
Blanc et al. [8]	Nicotine replacement therapies’ (NRT) used during pregnancy and the child’s health	Only a very limited number of papers have studied the safety and efficacy of NRT use during pregnancy on the child postnatal health. The paucity of existing data does not allow to draw conclusions. Further studies with adequate control groups should assess the use of NRT during pregnancy on their postnatal effects on the child health.
